# Supplementary material for: Should Lymph Nodes Be Retrieved in Patients with Intrahepatic Cholangiocarcinoma? A Collaborative Korea–Japan Study
Source: Cancers (Basel). 2021 Jan 25;13(3):445. doi: 10.3390/cancers13030445 (PMC7865580; doi:10.3390/cancers13030445)
Supplement: Supplementary file 1 [file cancers-13-00445-s001.pdf]

Should Lymph Nodes Be Retrieved in Patients with Intrahepatic Cholangiocarcinoma? A Collaborative Korea–Japan Study

Chang Moo Kang, Kyung-Suk Suh, Nam-Joon Yi, Tae Ho Hong, Sang Jae Park, Keun Soo Ahn, Hiroki Hayashi, Sae Byeol Choi, Chi-Young Jeong, Takeshi Takahara, Shigehiro Shiozaki, Young Hoon Roh, Hee Chul Yu, Takumi Fukumoto, Ryusei Matsuyama, Uyama Naoki, Kazuki Hashida, Hyung Il Seo, Takehiro Okabayashi, Tomoo Kitajima, Sohei SATOI, Hiroaki Nagano, Hongbeom Kim, Kaoru Taira, Shoji Kubo and Dong Wook Choi

Table S1. Operative findings of patients with resected IHCC.

	**Patients (*N* = 1138)**
Caudate Lobectomy	
No	934 (82.4%)
Yes	200 (17.6%)
Combined Resection	
No	383 (33.7%)
Yes	755 (66.3%)
Detailed Combined Resected Organs	
Gallbladder	695 (92.1%)
Stomach	4 (0.5%)
Duodenum	2 (0.3%)
Right adrenal gland	6 (0.8%)
Colon	4 (0.5%)
Small bowel	2 (0.3%)
Diaphragm	15 (2.0%)
Other	27 (3.6%)
Operation Time (min)	343.8 ± 147.3
Intraoperative Blood Loss (ml)	915.9 ± 1085.4
Intraoperative Transfusion	
No	659 (64.4%)
Yes	365 (35.6%)

Table S2. Pathologic findings of patients with resected IHCC.

**Mean ± SD**	**Patients (*N* = 1138)**
Radiologic tumor size (cm)	4.9 ± 3.2
Pathologic tumor size (cm)	5.1 ± 3.0
Retrieved lymph nodes	5.9 ± 8.3
Metastatic lymph nodes	1.1 ± 2.5
Gross type of tumor	
Mass forming	872 (76.6%)
Periductal infiltration	82 (7.2%)
Intraductal growth type	115 (10.1%)
Mixed	69 (6.1%)
Histopathologic grade	
Well differentiated	186 (17.8%)
Moderate differentiated	558 (53.3%)
Poorly differentiated	264 (25.2%)
Undifferentiated	38 (3.6%)
Portal vein invasion	
No	907 (81.9%)
Yes	201 (18.1%)

Mean ± SD	Patients (*N* = 1138)
Hepatic artery invasion	
No	1071 (96.7%)
Yes	37 (3.3%)
Lymphovascular invasion	
No	604 (58.2%)
Yes	434 (41.8%)
Perineural invasion	
No	666 (68.4%)
Yes	307 (31.6%)
Resection margin status	
R_0	1013 (89.0%)
R_1	106 (9.3%)
R_2	19 (1.7%)
Distance from resection margin (mm)	10.9 ± 14.3

Table S3. Topographic relationship between metastatic lymph nodes and primary tumor location of resected IHCC.

Tumor Side	Right-sided (*N* = 112)	Left-sided (*N* = 181)	*p*
No. 8 LN			0.121
No	85 (75.9%)	122 (67.4%)	
Metastasis	27 (24.1%)	59 (32.6%)	
No. 9 LN			0.998
No	104 (92.9%)	168 (92.8%)	
Metastasis	8 (7.1%)	13 (7.2%)	
No. 16 LN			0.485
No	100 (89.3%)	166 (91.7%)	
Metastasis	12 (10.7%)	15 (8.3%)	
Regional LN			0751
No	88 (78.6%)	145 (80.1%)	
Metastasis	24 (21.4%)	36 (19.9%)	
Peripancreatic LN			0.860
No	111 (99.1%)	179 (98.9%)	
Metastasis	1 (0.9%)	2 (1.1%)	

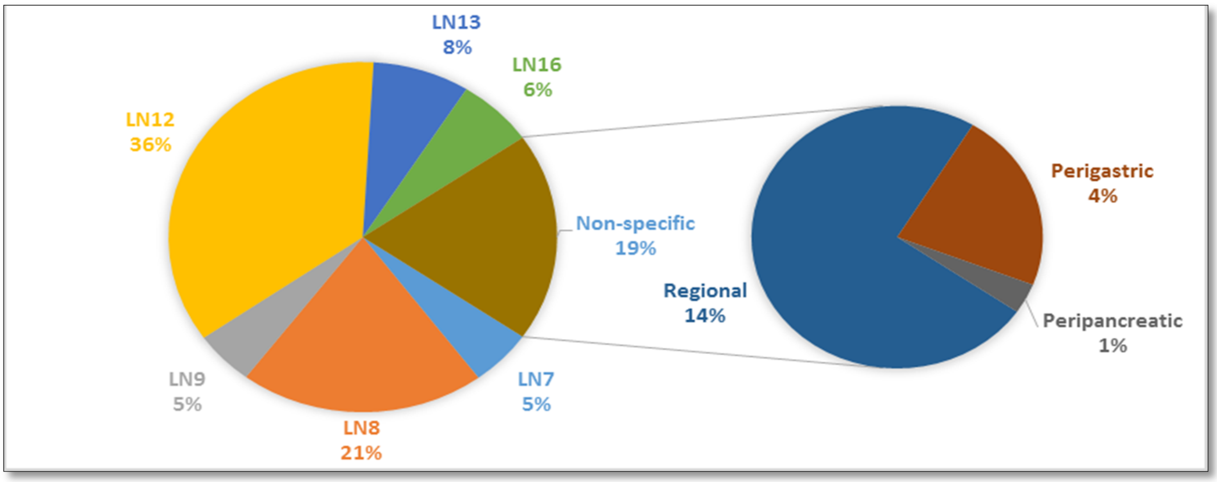

(**a**)

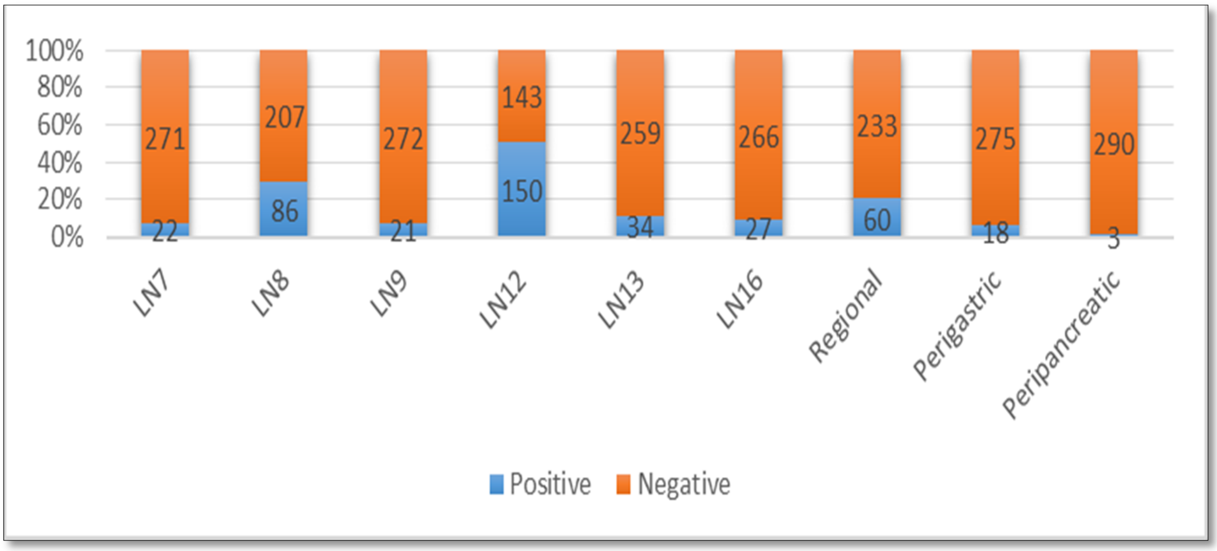

(**b**)

Figure S1. Status of metastatic and retrieved lymph nodes in resected IHCC. (**a**) Location of metastatic lymph nodes in IHCC (**b**) Proportion of metastatic lymph nodes according to the location of retrieved lymph nodes.

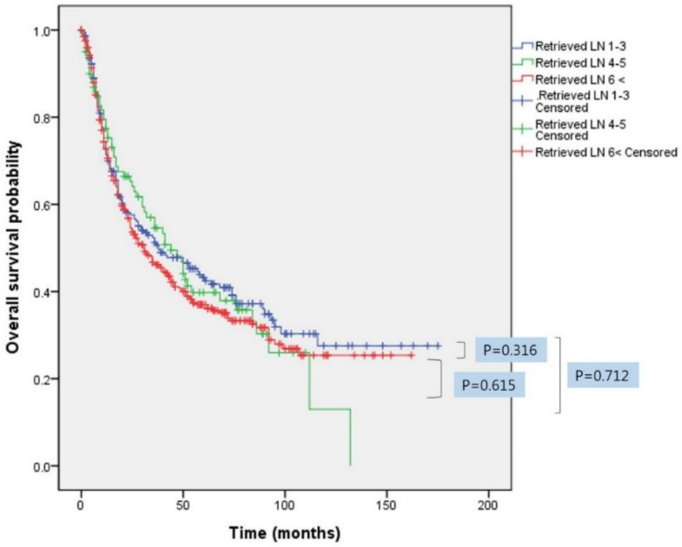

Figure S2. Overall impact of the number of retrieved lymph nodes in patients with resected IHCC.

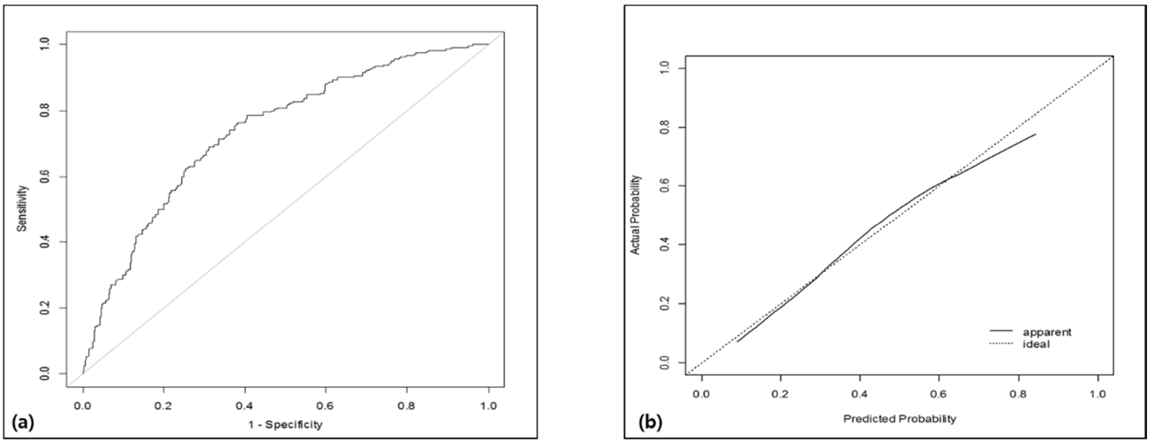

Figure S3. Accuracy of developed nomogram to preoperatively predict lymph node metastasis in patients with resected IHCC. (**a**) Internal validation of the nomogram. (**b**) Performance of the nomogram in the primary cohort.

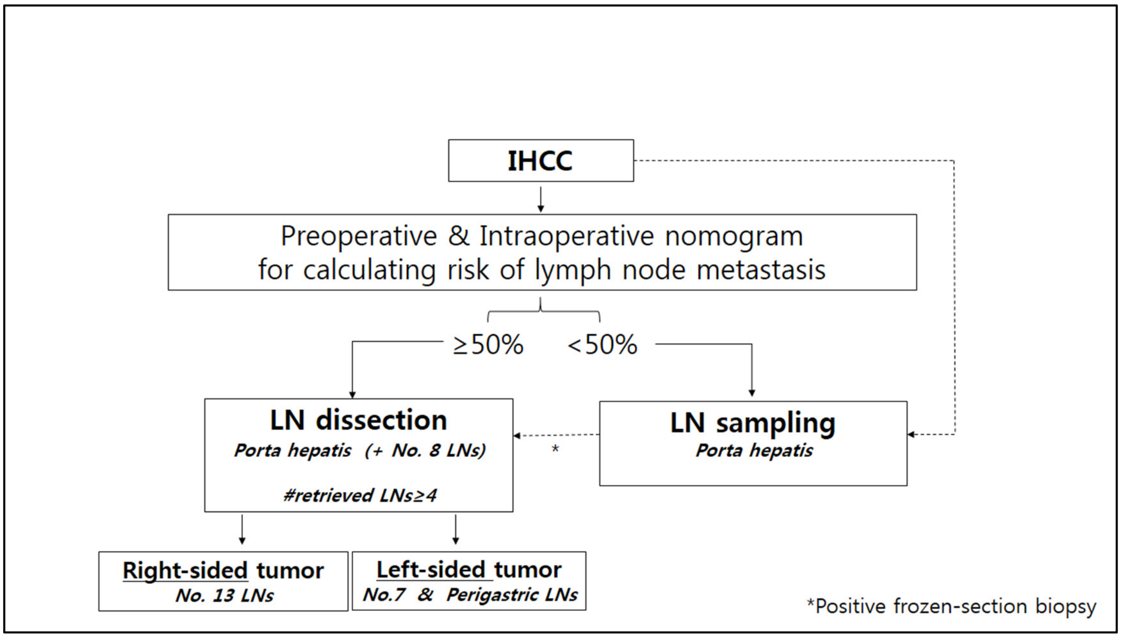

Figure S4. Proposal for case-specific surgical management of lymph nodes in treating IHCC.
